# Enhanced Cell Adhesion on Biofunctionalized Ti6Al4V Alloy: Immobilization of Proteins and Biomass from *Spirulina platensis* Microalgae

**DOI:** 10.3390/ijms27021041

**Published:** 2026-01-20

**Authors:** Maria Fernanda Hart Orozco, Rosalia Seña, Lily Margareth Arrieta Payares, Alex A. Saez, Arturo Gonzalez-Quiroga, Virginia Paredes

**Affiliations:** 1 Department of Mechanical Engineering, Universidad del Norte, Barranquilla 081007, Colombia; hartm@uninorte.edu.co; 2 Institute of Oncology Ángel Roffo, Faculty of Medicine, Universidad de Buenos Aires, Buenos Aires C1121A6B, Argentina; rosalia0719@gmail.com; 3 School of Engineering, RMIT University, Melbourne, Victoria 3001, Australia; s4148199@student.rmit.edu.au; 4 Biological Sciences and Bioprocess Group, School of Applied Sciences and Engineering, Universidad de EAFIT, Medellín 050022, Colombia; asaez@eafit.edu.co; 5 UREMA Research Unit, Mechanical Engineering Department, Universidad del Norte, Barranquilla 080001, Colombia; arturoq@uninorte.edu.co; 6 Biomedical Engineering Department, Universidad Simón Bolívar, Barranquilla 080002, Colombia

**Keywords:** Ti6Al4V alloy, surface biofunctionalization, cell adhesion, biomolecule immobilization, silanization (APTES), microalgae-derived coatings, *Spirulina platensis*

## Abstract

Titanium (Ti) and its alloys are widely used in biomedical applications due to their biocompatibility and corrosion resistance; however, surface modifications are required to enhance biological functionality. Surface functionalization using natural biomolecules has emerged as a promising strategy to improve early cell–surface interactions and biocompatibility of implant materials. In this study, Ti6Al4V alloy surfaces were biofunctionalized using *Spirulina platensis* (*S. platensis*) biomass and protein extract to evaluate morphological, chemical, and biological effects. The functionalization process involved activation with piranha solution, silanization with 3-aminopropyltriethoxysilane (APTES), and subsequent biomolecule immobilization. Surface characterization by scanning electron microscopy (SEM), inductively coupled plasma mass spectrometry (ICP-MS), energy-dispersive X-ray spectroscopy (EDS), and Fourier transform infrared spectroscopy (FTIR) confirmed the successful incorporation of microalgal components, including nitrogen-, phosphorus-, and oxygen-rich organic groups. Biomass-functionalized surfaces exhibited higher phosphorus and oxygen content, while protein-coated surfaces showed nitrogen-enrich chemical signatures, reflecting the distinct molecular compositions of the immobilized biomolecules. Cell adhesion assays demonstrated enhanced early cell attachment on biofunctionalized surfaces, particularly in samples functionalized with 5 g/L biomass for three hours, which showed significantly greater cell attachment than both the control and protein-treated samples. These findings highlight the complementary yet distinct roles of *S. platensis* biomass and protein extract in modulating surface chemistry and cell–material interactions, emphasizing the importance of tailoring biofunctionalization strategies to optimize early biological responses on titanium-based implants.

## 1. Introduction

Orthopedic implant materials must exhibit physical, mechanical, and biological properties comparable to bone to ensure proper implant–tissue interaction and long-term functionality, including osteointegration and bone remodeling upon contact with living tissues and biological fluids [1]. Titanium (Ti) is extensively used in orthopedic, dental, and cardiac implants due to its high biocompatibility and interaction with biological tissues, and excellent corrosion resistance [2,3,4]. However, implant rejection and inflammatory responses to this metal have been reported, highlighting the need to improve its biocompatibility. Consequently, surface modification strategies have been explored, as the implant surface is the primary interface with host tissue [3,5,6]. According to the “biocompatibility paradigm”, upon implantation, macromolecules, primarily proteins, adsorb onto the substrate. Physicochemical properties, surface energy, topography, and surface charge govern the protein-substrate interactions. Although this is a complex, multifactorial phenomenon, studies have reported that surface properties drive the adsorption of specific proteins, and that deliberate surface immobilization of biomolecules before implantation can enhance early biological responses, including cell adhesion and initial osteogenic signaling [7,8,9].

Biofunctionalization is a surface modification strategy that introduces or immobilizes biologically active molecules to create biomimetic coating. The process begins with surface activation or hydroxylation, generating reactive OH groups that facilitate the formation of chemical bonds with silanes (Metal-O-Si), and the subsequent immobilization of biomolecules. This modification enhances the surface ability to support protein adsorption and cell interactions, which are critical for initial biological recognition [10,11,12]. Several biomolecules have demonstrated enhanced biocompatibility, cellular adhesion, proliferation, and hydroxyapatite nucleation, thereby improving osteoblast adhesion [2,13]. Synthetic biomolecules, including proteins, peptide sequences, lipids, nucleic acids, carbohydrates, polysaccharides, and natural medicines, have been employed for surface immobilization [14,15,16]. It has been demonstrated that proteins can be immobilized on metals, and their adsorption is related to biocompatibility, corrosion, and wear properties of implantable metals [12,17]. They are considered a “biological cap” recognized by host cells [3]; however, these properties depend on the type of proteins, their concentration, and the surface characteristics of the metal [12].

Recent studies have explored biological molecules and co-culture systems to enhance surface biocompatibility and reduce cell detachment. Moreover, there is a growing need for environmentally friendly materials whose degradation byproducts are non-cytotoxic, which requires a complete understanding of the protein constituents involved [18]. Li et al. demonstrated that endothelial and smooth muscle cell co-culture promoted nitric oxide release, cell retention, and inhibition of hyperplasia, supporting the relevance of protein-functionalized surfaces in improving implant integration [19]. In orthopedic applications, covalent immobilization of bioactive molecules on titanium substrates has been shown to enhance protein adsorption and promote osteoblast attachment and proliferation [20]. Recent studies also indicate that immobilizing the KR12 peptide onto titanium alloys supports osteoblast cytocompatibility and antimicrobial activity [21], and that biofunctionalization with osteogenic proteins, such as BMP2, accelerates osseointegration and reduces prosthetic loading time [22], reinforcing the relevance of biomolecule-based surface functionalization strategies.

Microalgae are organisms found in marine and freshwater environments and are increasingly recognized as a sustainable source of bioactive compounds [23]. Approximately 50,000 species have been described, of which around 30,000 have been investigated [24]. These microorganisms can produce and accumulate a variety of biologically active biomolecules with antimicrobial, antioxidant, anticancer, anti-aging, and anti-inflammatory properties [25]. Their biomass is notably rich in protein, with some species such as *Spirulina* and *Chlorella* reaching 50–70% of their dry weight [26]. This high protein content, combined with a complete amino acid profile, makes microalgae attractive candidates for biomedical applications, including the surface biofunctionalization of implantable devices. In orthopedics, only a few studies have reported the application of microalgae for osteoinductive coating on metals, wound healing, drug delivery systems, and bone scaffolds [27,28]. This highlights their potential as a novel and eco-friendly platform for developing bioactive surfaces in regenerative medicine.

*Spirulina platensis* (*S. platensis*) is one of the most widely used microalgae in the food and pharmaceutical industries due to its rich nutritional profile and high levels of primary and secondary metabolites. Its biomass contains a significant proportion of proteins, essential fatty acids, vitamins, minerals, pigments, polysaccharides, and lipids, which position it as a candidate for therapeutic applications [26,29]. These constituents have demonstrated bioactivity against tumor-related diseases, inflammation, and bacterial and viral infections, and have also provided benefits for dietary supplementation [30,31]. Furthermore, *S. platensis* can be efficiently cultivated under industrial-scale conditions such as high solar exposure, moderate temperatures, and alkaline culture media, making it a sustainable source of biomolecules for biomedical applications, including the surface biofunctionalization of implantable devices [32].

However, the potential of microalgae—derived biomolecules for biofunctionalizing Ti6Al4V remains largely unexplored. It is hypothesized that immobilizing proteins or whole biomass from *S. platensis* onto Ti6Al4V surfaces can enhance their biocompatibility, particularly by improving early cell–surface interactions, as evidenced by enhanced in vitro cell adhesion. Based on this premise and considering that Ti-6Al-4V (Ti6Al4V) is the most commercially available Ti alloy for biomedical applications [33], this study aimed to biofunctionalize Ti6Al4V surface using both biomass and protein extracts from *S. platensis* and to assess their potential to enhance biocompatibility, as determined by in vitro cell adhesion assays. The methodology involved surface activation with piranha solution, silanization with APTES (3-aminopropyltriethoxysilane), and subsequent immobilization of microalgal biomolecules, followed by comprehensive physical and chemical characterization using scanning electron microscopy (SEM), Inductively Coupled Plasma Mass Spectrometry (ICP-MS), Energy Dispersive X-ray Spectroscopy (EDS), Fourier Transform Infrared Spectroscopy (FTIR), and cellular assays. Silanization with APTES was incorporated as a key intermediate step to generate an amine-terminated silane layer covalently anchored to surface hydroxyl groups, promoting stable and homogeneous immobilization of proteins and biomass through Ti–O–Si linkages and intermolecular interactions.

Given that biomolecule-based coatings are inherently sensitive to preservation and sterilization processes, which may compromise the structural integrity and activity of proteins and polysaccharides [34], the biofunctionalized surfaces in this study were used immediately under sterile conditions. This limitation is acknowledged as part of a proof-of-concept approach, and future developments will require evaluation of storage stability and sterilization-compatible strategies to support potential translational applications.

## 2. Results

### 2.1. Stage 1: Activation

**Figure 1:**
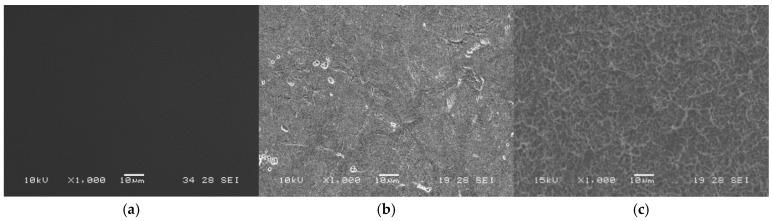
Scanning electron microscopy (SEM) micrographs of Ti6Al4V surfaces: (**a**) control sample, (**b**) activated with a 3:1 piranha solution, (**c**) activated with a 1:1 piranha solution.

The SEM micrographs in Figure 1 reveal morphological changes on the Ti6Al4V surface after the activation treatment. The polished surface (Figure 1a) displays a uniform morphology, while the surfaces treated with piranha solution (H_2_SO_4_:H_2_O_2_) at ratios of 3:1 and 1:1 (Figure 1b and Figure 1c, respectively) exhibit topographical irregularities attributable to chemical exposure. An enhancement in surface textural features was observed in the specimen treated with the 1:1 piranha solution, attributable to the higher density of micro-pitting generated during the treatment. This effect is less pronounced in the specimen treated with the 3:1 piranha solution, as evidenced by the SEM images.

The concentration of active hydroxyl groups (C_OH_) was estimated using the zinc complex substitution technique [35]. The values, normalized by area and presented in Table 1, showed an increase in the number of OH groups on all activated surfaces compared to the polished control (827.88 OH act/nm^2^). The highest concentration of OH groups was observed in the sample treated with the 1:1 solution, confirming a more effective activation.

### 2.2. Stage 2: Silanization

**Figure 2:**
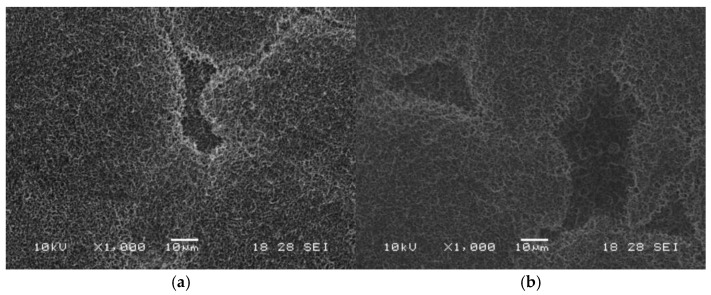
SEM micrographs of silanized samples after (**a**) 1 h and (**b**) 3 h of silanization.

The SEM images in Figure 2 reveal the formation of a thin, irregular film on the titanium surface following silanization, observed for both immersion durations (1 h and 3 h). The film, depicted as white and gray regions, suggests silane deposition and the presence of zones with reduced surface coverage.

**Figure 3:**
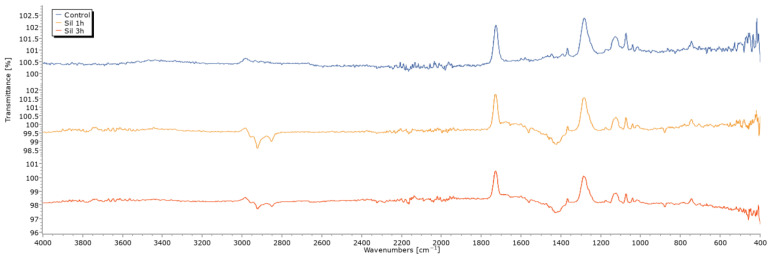
Fourier Transform Infrared Spectroscopy (FTIR) spectra of control sample and samples silanized for 1 h and 3 h.

These morphological changes were further explored via FTIR spectroscopy (Figure 3). Although a very weak feature can be perceived near 3700 cm^−1^, its intensity is comparable to baseline fluctuations and >100% transmittance artifacts, and therefore, it cannot be considered solid evidence of free O–H groups. The region between 2800–3000 cm^−1^ shows C–H stretching signals consistent with the presence of organic moieties from APTES) [36]. A band is also observed near 1558 and 1425 cm^−1^; however, this vibration is not uniquely diagnostic of primary amine N–H bending from APTES and may include contributions from amide II, H–O–H bending, or other mixed C–N/N–H modes. The silanized sample at 1 h showed stronger signals; this apparent increase may reflect instrumental or sampling variability. Thus, although a modification in the spectrum is observed, these differences cannot be interpreted as higher surface coverage, as FTIR band intensity may vary due to factors unrelated to chemistry, such as ATR contact, sample thickness, or baseline artifacts. A band at 800–900 cm^−1^, associated with the formation of Ti–O–Si bonds, was also detected [37].

EDS analysis (Table 2) confirmed the presence of silicon in both silanized samples at low levels (~0.5 wt%), consistent with the formation of the APTES layer. The corresponding atomic percentages are low, as expected for a thin organosilane overlayer and for EDS measurements influenced by ZAF corrections. The differences observed in Ti, Al, and V signals between the control and the silanized samples are expected for modified surfaces and arise from the combined effects of partial coverage by the organosilane layer, the shallow penetration depth of EDS, and spot-to-spot variability associated with local surface topography. These factors reduce the effective contribution of the metallic substrate to the detected spectrum and explain the apparent compositional changes that do not correspond to modifications of the bulk alloy [38]. The EDS profiles obtained after 1 h and 3 h of immersion exhibited comparable trends, and both conditions were therefore interpreted as providing similar levels of surface functionalization.

### 2.3. Stage 3: Immobilization of S. platensis Biomass and Protein Extract

**Figure 4:**
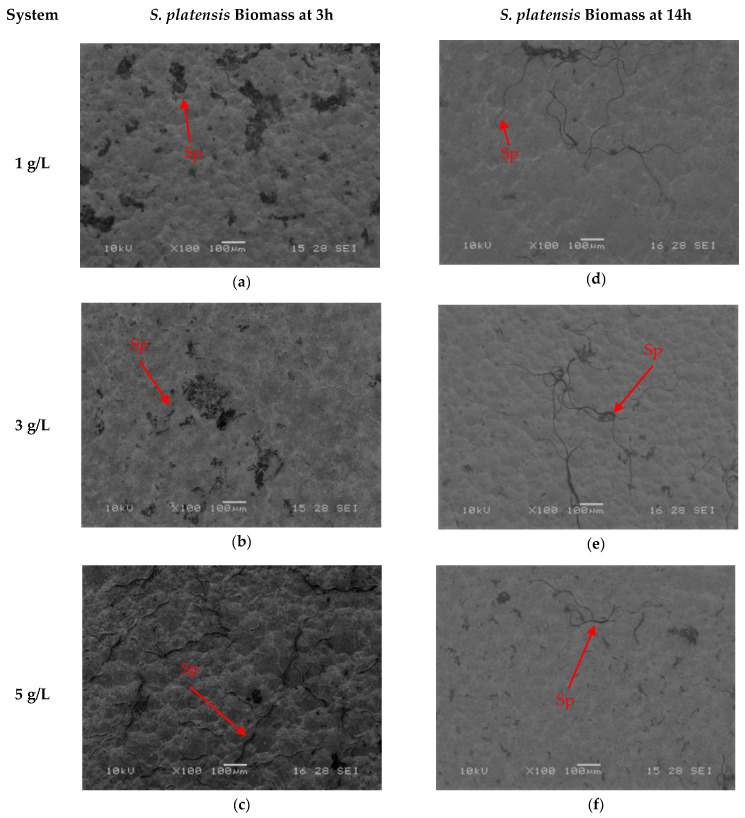
SEM micrographs of samples with immobilized *S. platensis* biomass at different concentrations and immersion times: (**a**) 1 g/L, 3 h; (**b**) 3 g/L, 3 h; (**c**) 5 g/L, 3 h; (**d**) 1 g/L, 14 h; (**e**) 3 g/L, 14 h; (**f**) 5 g/L, 14 h.

**Figure 5:**
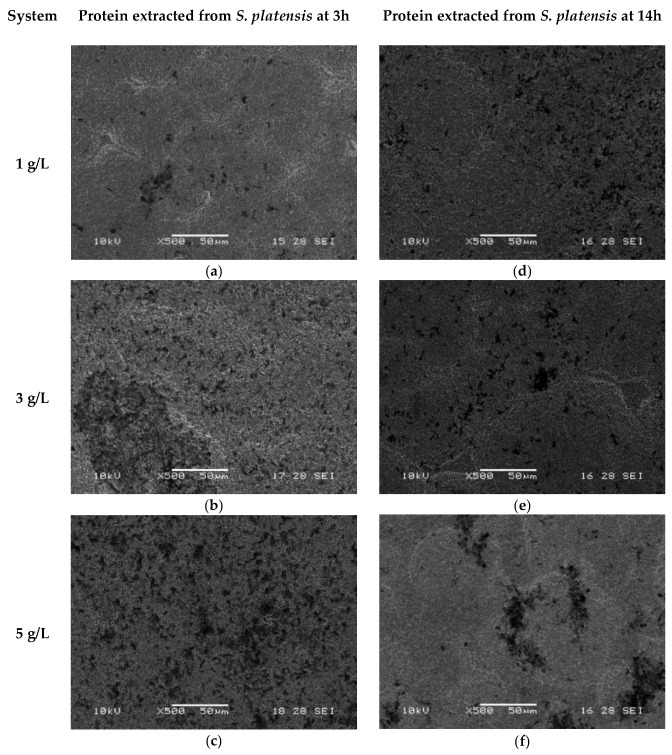
SEM micrographs of samples after immobilization of *S. platensis* protein extract at different concentrations and immersion times: (**a**) 1 g/L, 3 h; (**b**) 3 g/L, 3 h; (**c**) 5 g/L, 3 h; (**d**) 1 g/L, 14 h; (**e**) 3 g/L, 14 h; (**f**) 5 g/L, 14 h.

SEM micrographs shown in Figure 4 and Figure 5 illustrate the surface morphology after the immobilization of *S. platensis* biomass and protein extract for two durations (3 h and 14 h). In the biomass-treated samples (Figure 4a–f), spiral-shaped microalgal structures were observed distributed across the titanium surface, with the highest coverage recorded in the samples treated for 3 h at a concentration of 5 g/L (Figure 4c). Progressive coverage was also observed at 3 g/L and 1 g/L (Figure 4a and Figure 4b, respectively). For the 14 h treatments (Figure 4d–f), no significant increase in coverage was observed, and in some cases, a lower amount of adhered biomass was detected.

In contrast, the surfaces treated with the protein extract (Figure 5a–f) showed irregular dark regions, suggesting the presence of protein aggregates distributed in island-like clusters. At higher protein concentration (5 g/L) and shorter immersion time (3 h, Figure 5c), a denser and more uniform coverage was observed, while concentrations of 3 g/L and 1 g/L (Figure 5a,b) showed a progressive increase in coverage. Samples treated for 14 h (Figure 5d–f) exhibited less homogeneous distribution.

**Figure 6:**
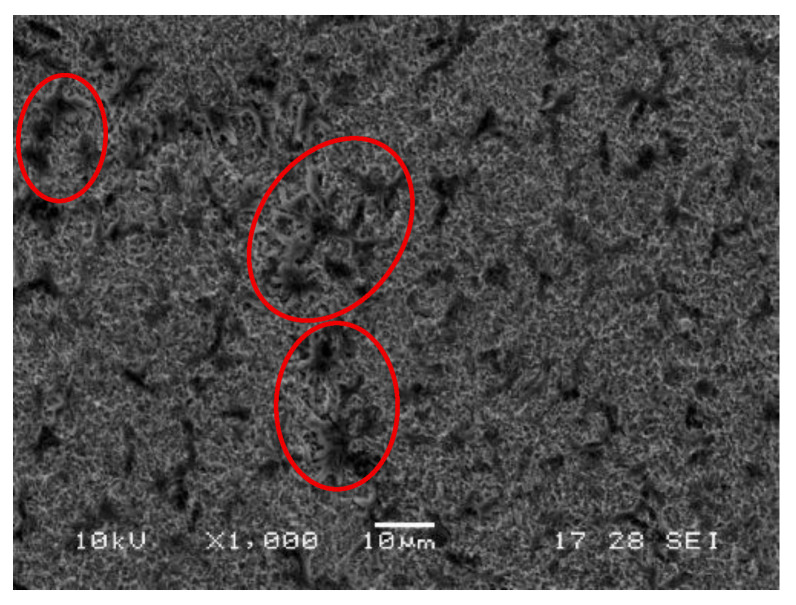
Higher-magnification SEM image of the sample treated with protein extract (5 g/L, 3 h), the red ovals highlight representative clusters of protein aggregates immobilized on the silanized titanium surface.

Figure 6 shows a higher-magnification view in which the protein clusters on the functionalized surface are clearly visible after treatment with protein extract at 5 g/L for 3 h.

**Figure 7:**
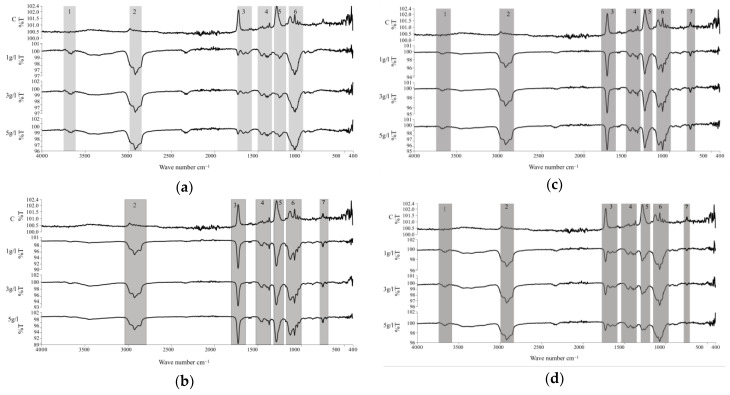
FTIR spectra of samples after immobilization with *S. platensis* biomass and protein extract at different immersion times: (**a**) biomass, 3 h; (**b**) biomass, 14 h; (**c**) protein extract, 3 h; (**d**) protein extract, 14 h.

The infrared spectra of the samples after immobilization of *S. platensis* biomass and protein extract are presented in Figure 7, and the corresponding functional groups are summarized in Table 3. Spectral differences were observed between the silanized and biofunctionalized samples. In three of the four spectra analyzed, six main regions were identified, associated with functional groups present in proteins, lipids, fatty acids, and polysaccharides [39]. The peak in region 1 (3700 cm^−1^) corresponds to hydroxyl groups (O–H) generated during the piranha activation step [40]. Bands in region 2 (3000–2800 cm^−1^) and region 3 (1750–1700 cm^−1^) are attributed to aliphatic C–H stretching and C=O stretching vibrations, typically associated with lipids, esters, and carboxylic acids [41]. Within region 3 (1670–1610 cm^−1^), a well-defined band corresponding to the amide I mode was observed, indicative of protein secondary structures such as α-helices and β-sheets [42].

Region 4 (1460–1360 cm^−1^) revealed symmetric methyl (CH_3_) bending modes, which are also linked to protein structures [43]. Region 5 (1270–1230 cm^−1^) included bands corresponding to phosphodiester stretching, possibly originating from nucleic acids or phosphorylated proteins [44]. Finally, region 6 (1200–1000 cm^−1^) displayed bands corresponding to C–O–C stretching in glycosidic linkages and ring vibrations, characteristic of polysaccharides with substituents such as phosphate, sulfonate, or carboxymethyl. In addition, a broad but consistent band was observed in region 7 (750–650 cm^−1^), which is attributed to out-of-plane =C–H bending modes associated with aromatic structures naturally present in *S. platensis* biomass [43].

Overall, the FTIR analysis confirms the presence of organic functional groups associated with the immobilized biomolecules, clearly differentiating the biofunctionalized surfaces from the silanized and control samples.

**Table 3:** Assignment of functional groups and structures observed in the FTIR spectra.

Region	Group	Range (cm^−1^)	Type de Vibration	Ref.
1	Hydroxyl	3700	O-H	[40,45]
2	Aliphatic—CH_2_	3000–2800	C-H aliphatic stretching	[41]
3	Lipids and fatty acid esters	1750–1700	C=O Vibratory stretching	[43]
	Proteins	1670–1610	C=O stretching and bending vibration of NH	[46]
4	Proteins	1460–1360	Symmetrical bending of CH_3_	[43]
5	Phosphodie-sters	1230–1270	P=O	[44]
6	Polysaccharides and substituent groups	1200–1000	P–O stretching of phosphate groups, C–O–C stretching of glycosidic bonds and polysaccharide ring vibrations	[36,43]
7	Aromatic =C–H	750–650	Out-of-plane =C–H bending from aromatic structures	[43]

EDS analysis results after immobilization of *S. platensis* biomass and protein extract are shown in Table 4. All biofunctionalized samples exhibited higher atomic carbon percentages, as well as the presence of oxygen, nitrogen, and phosphorus, compared to the silanized samples (Table 2). These elements are inherent to the molecular composition of cyanobacteria, confirming the effective deposition of microalgal biomolecules on the titanium surface [47,48].

Elemental values are reported as mean ± SD based on replicate measurements and are presented for descriptive purposes. Biofunctionalized samples exhibited increased carbon-related signals compared to silanized surfaces, with higher carbon intensities observed at longer immersion times (14 h) relative to shorter treatments (3 h), indicating a greater accumulation of organic material on the surface. Trends in elemental signals varied with biomolecule type: biomass-treated samples showed comparatively higher oxygen-related signals, while protein-treated samples exhibited increased nitrogen-related signals, consistent with their respective biochemical compositions [49]. Phosphorus was detected only in biomass-treated samples, likely due to the presence of membrane phospholipids, which are absent in the protein extract as a result of the extraction and filtration process [34,50]. Regarding metallic elements (Ti, Al, V), a relative decrease was observed in the biomass-treated specimens, suggesting a more extensive and uniform surface coverage under these conditions.

EDS values are reported as mean ± SD from replicate measurements. Given the semi-quantitative nature of EDS and the unavailability of raw spectral and mapping files, the interpretation focuses on elemental detection trends rather than spatial distribution.

### 2.4. Stage 4: Cell Adhesion Assay

**Figure 8:**
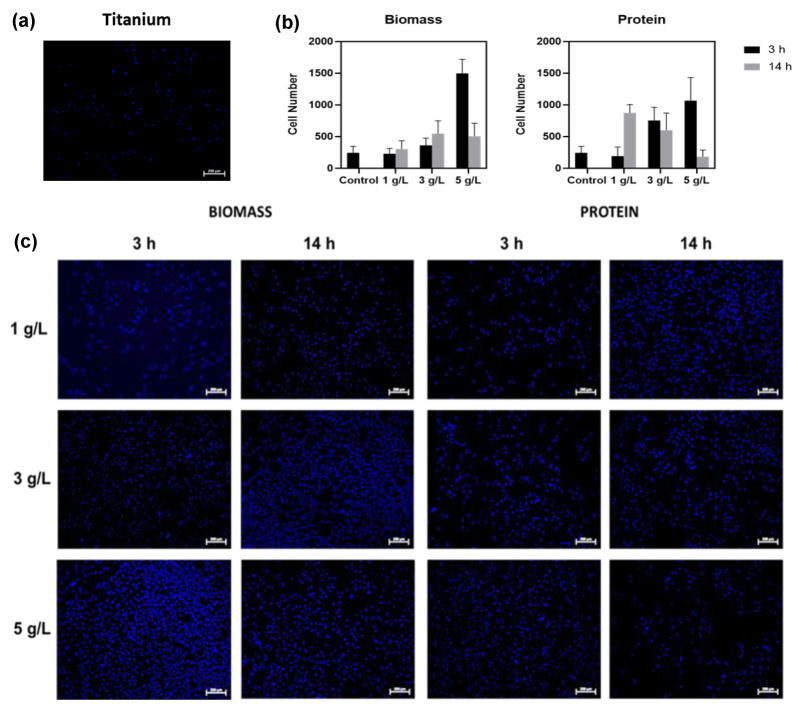
DAPI-fixed cells after 24 h of incubation. (**a**) Cells adhered to titanium base, (**b**) Number of cells adhered in each of the treatments, after 24 h of incubation, (**c**) Cells stained with DAPI adhered to biofunctionalized titanium with biomass and protein from the microalgae *S. platensis*, in three different concentrations and at two times of immobilization of microalgae and proteins. Scale bar = 200 μm.

Cell adhesion assays were conducted on titanium substrates biofunctionalized with *S. platensis* biomass (TiB) and protein extract (TiP) at three concentrations (1, 3, and 5 g/L) and two immobilization durations (3 h and 14 h). Quantitative results are reported as mean ± SD. As shown in Figure 8a,b, the TiB group exhibited a statistically significant increase in the number of adhered cells as the biomass concentration increased (*p* < 0.001). All TiB samples demonstrated significantly higher cell adhesion compared to the control, except for the sample functionalized with 1 g/L biomass for 3 h, which showed no statistically significant difference relative to the untreated substrate.

Regarding immobilization time, TiB samples treated for 14 h showed improved cell adhesion at 1 g/L and 3 g/L compared to those treated for 3 h counterparts. However, at the 5 g/L concentration, cell adhesion decreased when the immobilization time increased from 3 h to 14 h (Figure 8b). Two-way ANOVA revealed a significant interaction between concentration and immobilization time (*p* < 0.05).

In the TiP group, a progressive increase in cell adhesion was observed with increasing protein concentration during 3 h treatments (Figure 8b,c). For the 14 h samples, although some variability was noted across concentrations, all protein-functionalized conditions exhibited significantly more adhered cells than the control surface. Notably, none of the TiP groups outperformed the TiB 5 g/L–3 h sample, which registered the highest cell density among all tested conditions.

These results indicate that both the type of biomolecule and the parameters of the immobilization process, namely, concentration and duration, play a critical role in modulating cellular response. Optimized conditions, particularly the TiB sample treated for 3 h at 5 g/L, yielded the most favorable cell adhesion outcomes.

## 3. Discussion

### 3.1. Stage 1: Activation

The morphological modification of Ti6Al4V induced by piranha treatment is associated with its aggressive chemical action, which produces prominent surface features, particularly at the 1:1 ratio, as indicated by qualitative observations of SEM images. This type of topography has been reported to be favorable for cell surface interactions, as rough and hydrophilic surfaces can promote early cell adhesion and spreading [51]. The assessment of OH group concentration, which provides a quantitative indicator of surface hydroxylation, showed that both piranha treatments enhanced the availability of these functional groups. However, the 1:1 solution not only significantly increased the presence of hydroxyl groups but also produced more uniform and reproducible morphological changes. These conditions favor the formation of Metal-O-Si bonds during silane coupling [52]. Therefore, the 1:1 activation condition was selected as the most suitable for the subsequent functionalization stages based on the combined optimization of surface chemistry and morphology.

### 3.2. Stage 2: Silanization

The SEM, FTIR, and EDS findings confirm the effectiveness of the silanization process in inducing both physical and chemical modifications on the activated titanium surfaces. SEM micrographs revealed the formation of a thin, irregular film after APTES treatment, indicating silane deposition. This morphological change aligns with previous studies suggesting that short immersion times and low APTES concentrations promote the formation of thin and uniform silane monolayers, minimizing undesired polymerization [53].

FTIR chemical characterization further supported the presence of the silane layer, with distinct bands corresponding to O–H, C–H, and N–H vibrations. Although variations in band intensity were observed between the silanized samples, these differences should be interpreted cautiously, as FTIR signal intensity can be influenced by factors such as ATR contact, surface roughness, and baseline effects. Nonetheless, the detection of characteristic C–H and N–H vibrations confirms the successful introduction of organosilane functionalities on the titanium surface. The appearance of the Ti–O–Si vibration band in the 800–900 cm^−1^ region also confirms successful chemical anchoring between the titanium substrate and the silane agent, formed by substitution of Ti–O–H groups generated during activation [38].

EDS analysis showed the incorporation of silicon under both silanization conditions, in line with the expected elemental profile of APTES. The low silicon content detected is consistent with the presence of a thin organosilane overlayer rather than a thick polymerized film. Considering the comparable elemental profiles obtained for the two immersion times and the more homogeneous surface morphology observed in the 1 h treatment, this condition was selected for subsequent biofunctionalization and biological evaluation. This choice is consistent with literature which emphasizes the benefits of mild silanization protocols for preserving the reactivity of terminal functional groups and improving molecular orientation [46,53].

### 3.3. Stage 3: Immobilization of S. platensis Biomass and Protein Extract

The SEM analysis confirmed successful immobilization of *S. platensis* biomass and protein extract on silanized titanium surfaces, with marked differences in surface coverage and distribution patterns between the two types of biomolecules. Biomass-coated samples revealed spiral-shaped microalgal structures, indicative of the intact morphology of *S. platensis*, particularly in samples treated for 3 h at 5 g/L (Figure 4c). The higher coverage observed under this condition aligns with the expected behavior of intact cyanobacteria, whose larger structures enhance topographic surface occupancy and may promote multivalent interactions with the silanized substrate [54].

In contrast, protein-coated samples exhibited island-like aggregates that were more homogeneously distributed across the surface (Figure 5a–c), especially at 3 h and 5 g/L (Figure 5c). This pattern can be attributed to the absence of cellular scaffolding and the greater molecular mobility of the extracted proteins during the immobilization process, facilitating broader surface interactions and more uniform deposition, as suggested by the increased clustering observed under high magnification (Figure 6).

FTIR spectra further supported these morphological differences by revealing characteristic signals associated with the chemical nature of both proteinaceous and lipidic components. The presence of O-H stretching at 3700 cm^−1^ and overlapping peaks in regions 2 and 3, including C–H and C=O stretching modes, corroborated the coexistence of lipid and protein fractions in the immobilized layer [40,41]. Notably, the amide I band (1670–1610 cm^−1^), a sensitive marker for protein secondary structure, was clearly defined in the protein-treated samples, suggesting partial preservation of native α-helices and β-sheet conformations upon immobilization [42]. These features are critical in maintaining the bioactivity of immobilized proteins, particularly in applications requiring cell–material interaction [17,41,42].

Additional insights were provided by signals detected in regions 4 to 6 of the FTIR spectra. The CH_3_ bending modes (1460–1360 cm^−1^) and phosphodiester bands (1270–1230 cm^−1^) confirmed the presence of protein-associated structures and nucleic acid residues [44]. Meanwhile, the complex vibrational patterns in region 6 (1200–1000 cm^−1^), including C–O–C and glycosidic ring modes, are indicative of polysaccharides bearing phosphate, sulfonate, or carboxymethyl substituents [43].

Furthermore, signals observed in region 7 (750–650 cm^−1^) are associated with out-of-plane skeletal vibrations and deformation modes of polysaccharide backbones and phosphate-containing groups, which are commonly reported for complex biopolymers derived from microalgal biomass. Although these bands are typically less intense and more overlapped, their presence further supports the incorporation of polysaccharidic and phosphorylated components from *S. platensis* within the immobilized layer [43]. These findings emphasize the intricate macromolecular composition of *S. platensis*, whose matrix comprises proteins, nucleic acids, and polysaccharides, offering multiple binding moieties for surface interactions and functionalization [44]

Consistent with FTIR results, EDS analysis revealed increased levels of carbon, nitrogen, oxygen, and phosphorus on functionalized surfaces compared to silanized controls (Table 4), confirming the deposition of organic material derived from *S. platensis* [47,48]. Although higher carbon levels were detected after 14 h treatments, this increase reflects cumulative surface accumulation rather than indicating changes in the bulk alloy composition; as evidenced by the more effective coverage at 3 h. Biomass-treated samples showed higher oxygen content than protein-treated ones, which may be attributed to the presence of lipid and polysaccharide components rich in oxygenated groups. In contrast, nitrogen content was more pronounced in protein-treated samples, consistent with the high nitrogen content of amino acids and peptide bonds [49].

The absence of phosphorus in protein-coated samples may be explained by the extraction process, which removes membrane-bound phospholipids and other phosphorylated residues through filtration [34,50], while the retention of phosphorus in biomass-treated samples likely originates from the phospholipid-rich membranes of intact cyanobacterial cells [55]. The reduced detection of metallic elements such as titanium and aluminum in biomass-coated samples further supports the presence of a more uniform and continuous organic layer, rather than changes in the bulk alloy composition, suggesting improved surface coverage compared to protein-coated substrates.

Together, these findings confirm that both *S. platensis* biomass and protein extract effectively bind to silanized titanium, each providing distinct morphological and chemical surface properties. It should be noted that EDS results are interpreted qualitatively, given the semi-quantitative nature of the technique, and therefore are not used to infer uniform surface coverage or statistically significant compositional differences. The greater molecular complexity of the biomass appears to confer more extensive coverage and retention of diverse functional groups, whereas the protein extract enables more controlled and homogeneous deposition, albeit with reduced macromolecular diversity. This highlights the need to tailor surface biofunctionalization strategies according to the specific biological or mechanical demands of the intended biomedical application.

### 3.4. Stage 4: Cell Adhesion Assay

The results of the cell adhesion assays indicate that both *S. platensis* biomass and protein extract enhance the biocompatibility of titanium surfaces [56]. Samples biofunctionalized with biomass showed a statistically significant increase in cell density with increasing concentration, highlighting the role of biomolecular load in promoting initial adhesion. However, in the 5 g/L TiB samples treated for 14 h, a decrease in cell adhesion was observed compared to their 3 h counterparts, suggesting a saturation or alteration of the surface coating over prolonged exposure. This behavior may be related to the lower immobilization efficiency seen in SEM images (Figure 4), which could limit the bioavailability of surface-bound cues for cell interaction. It is important to emphasize that the biological evaluation performed in this study was specifically designed to assess early-stage cell adhesion and surface biocompatibility, rather than osteogenic differentiation or long-term osseointegration.

In this context, the use of Vero CCL-81 cells provides a robust and reproducible model for screening cell–surface interactions, allowing the isolation of surface-driven effects on initial cell attachment independently of lineage-specific differentiation pathways.

These findings align with previous studies reporting that extended immobilization times may lead to excessive deposition, structural rearrangements, or even surface masking, thereby reducing the accessibility of active biomolecular groups essential for cell attachment [57]. Moreover, increasing cell confluence over time can result in changes in morphology, cytoskeletal organization, and intercellular interactions, which may alter adhesion behavior and limit further cell attachment as the surface becomes saturated. These observations are consistent with experimental and simulation data indicating that high cell densities can significantly modify cellular interaction dynamics and distribution patterns [58].

In contrast, surfaces functionalized with protein extract (TiP) showed a more uniform island-like distribution of adhered cells, with improved adhesion observed at higher concentrations and shorter immobilization times (3 h). Although all TiP conditions led to greater cell adhesion than the control, none surpassed the adhesion observed in TiB at 5 g/L for 3 h, the condition that yielded the highest cell density. These results agree with reports by Mohd Talha et al. and others [10,11,12], who demonstrated that protein coatings enhance the initial cell response to metallic surfaces by facilitating biomolecule-mediated attachment. The more homogeneous distribution observed in TiP systems may be attributed to the absence of cellular structures and higher molecular mobility during protein immobilization, which allows for more even surface coverage.

Taken together, these results highlight the importance of optimizing both biomolecule concentration and immobilization time to obtain bioactive coatings with reproducible biological performance. Biomass-functionalized surfaces not only exhibited higher oxygen content but also retained phosphorus—elements known to support osteogenic activity. However, it is important to note that early cell adhesion does not exclusively depend on phosphate chemistry and should not be interpreted as a direct predictor of long-term osteointegration outcomes. This distinction was particularly evident in the TiB 5 g/L for 3 h condition. In contrast, TiP samples were enriched in nitrogen, a key component of protein structures, and this enrichment correlated with improved adhesion compared with the untreated control. In this context, the multifunctionality of *S. platensis*, which includes antioxidant and antimicrobial compounds, positions it as a promising candidate for developing bioactive and protective implant coatings [59].

Although phosphate-containing groups are widely associated with osseointegration, early cell adhesion is not governed exclusively by surface phosphorus content. Recent studies have demonstrated that initial cell–surface interactions are strongly regulated by surface chemistry, topography, hydrophilicity, and the nature of adsorbed organic molecules, which modulate integrin-mediated signaling pathways and cytoskeletal organization. In this context, the enhanced adhesion observed for the Biomass 5 g/L and Protein 5 g/L conditions can be plausibly explained by the combined effects of activation-induced surface hydroxylation, favorable micro- and nanoscale topographical features observed by SEM, and the presence of protein- and polysaccharide-related functional groups identified by FTIR. These synergistic factors provide bioactive cues that promote early cell attachment independently of phosphate chemistry, supporting the interpretation that the improved adhesion response arises from the multifunctional organic interface generated by the microalgal-derived coatings rather than from elemental composition alone [60].

Additionally, a recent study by Castillejo et al. and Peng et al. highlighted the role of natural biomolecule-based coatings, such as those incorporating chitosan, nanohydroxyapatite, polydopamine, and antimicrobial peptides, not only in promoting osteoblast adhesion but also in providing antibacterial functionality [61,62]. These findings reinforce the potential of microalgae-derived coatings to mimic extracellular matrix signals and simultaneously introduce chemical and topographical cues that enhance cell–substrate interactions. Additional support is provided by Cicco et al. [63], who demonstrated improved cytocompatibility and cytoskeletal reorganization on biosilica-coated substrates, a phenomenon similarly observed in this study with *S. platensis*-based coatings. This suggests that the morphological and biochemical modifications induced by microalgal coatings promote stronger cellular anchorage and cytoskeletal organization, key factors in osteointegration.

Altogether, the results of this study indicate that coatings derived from *S. platensis* function not only as biocompatible interfaces but also as potential bioactive matrices capable of guiding early cell adhesion and supporting tissue integration. However, it should be noted that early cell adhesion does not necessarily predict long-term osteointegration, which is a multifactorial process involving osteogenic differentiation, extracellular matrix deposition, and bone remodeling.

Accordingly, future studies should incorporate osteoblasts or mesenchymal stem cells, as well as proliferation, differentiation, and mineralization assays, to further elucidate the osteoinductive potential of these microalgae-derived biofunctionalized surfaces.

## 4. Materials and Methods

### 4.1. Materials

Commercial Ti6Al4V alloy discs (8 mm in diameter and 5 mm in thickness) were used. Each specimen was sequentially polished with SiC paper (60, 240, 320, 600, and 2000 grit), followed by washing in an ultrasonic bath for 6 min each in deionized water, 96% ethanol, and acetone [3].

### 4.2. Biofunctionalization of the Surface

#### 4.2.1. Surface Activation with Piranha Solution

Surface activation was performed using piranha solution prepared by mixing 98% H_2_SO_4_ and 30% H_2_O_2_ at volume ratios of 3:1 (Act 3:1) and 1:1 (Act 1:1). Piranha is an oxidant capable of generating hydroxyl groups on the Ti surface, making it hydrophilic [64]. The specimens were immersed in piranha solution for 1 h at room temperature. Each condition was performed in triplicate to ensure reproducibility. After activation, samples were rinsed thoroughly with deionized water before silanization.

#### 4.2.2. Silanization with APTES

Silanization was performed by immersing the activated specimens in a 1:20 (*v*/*v*) solution of 3-aminopropyltriethoxysilane (APTES) in anhydrous toluene at 100 °C. Two immersion times were evaluated: 1 h (Sil1h) and 3 h (Sil3h) to determine which produced better results regarding Si presence and the homogeneity of the silane on the surface. Following silanization, the process specimens were ultrasonically cleaned in deionized water, 96% ethanol, and acetone, and then air-dried under sterile conditions before biomolecule immobilization [10,65].

#### 4.2.3. Immobilization of Biomolecules

The biomolecules used for immobilization were *S. platensis* biomass and protein extract. Immobilization was achieved by immersing the silanized specimens in *S. platensis* biomass or protein extract solutions at concentrations of 1 g/L, 3 g/L, or 5 g/L for 3 h or 14 h under constant agitation at 200 rpm and room temperature. After immersion, the specimens were rinsed sequentially with phosphate-buffered saline (PBS) and deionized water and then air-dried under sterile conditions. The protein extract was obtained from *S. platensis* using a deep eutectic solvent (DES)-based extraction method (choline chloride–glycine, 1:2 molar ratio), followed by incubation at 45 °C under shaking, centrifugation, dilution in deionized water, and filtration through 0.45 µm syringe filters before immobilization [66]. Biomass immobilization was performed directly from a sterile *S. platensis* culture suspended in deionized water.

### 4.3. Surface Characterization

#### 4.3.1. Morphological Characterization of the Surfaces

The morphological characterization of the base substrate, polished, activated, silanized, and biofunctionalized surfaces was performed using scanning electron microscopy (SEM, Carl Zeiss EVO MA10, Zeiss, Jena, Germany). Observations were performed at accelerating voltages of 10 and 15 kV, with a working distance of 28 mm and magnifications ranging from 100× to 1000×. Each sample was analyzed in triplicate to ensure reproducibility.

#### 4.3.2. Chemical Characterization of the Surfaces

The effectiveness of the activation process was evaluated using the zinc complex substitution method described by Sakamoto et al. [35]. Activated specimens were immersed for 5 min in a 0.10 M zinc complex solution (containing 0.10 M ZnCl_2_ and 2.0 M NH_4_Cl, adjusted to pH 6.9 NH_4_OH) to form zinc complexes on the surface oxide layer. Subsequently, the specimens were rinsed with ethanol and dried in a desiccator for 1 h. Then they were immersed in nitric acid to release the zinc ions into the solution.

The concentration of released ions was determined by Inductively Coupled Plasma Mass Spectrometry (ICP-MS, ICAP 7000 Series, Thermo Scientific, Waltham, MA, USA). For ICP-MS analysis, all samples were digested in closed pressurized vessels using a microwave digestion system (MARS Xpress, CEM Corporation, Matthews, NC, USA) equipped with a 40-position turntable. ICP-MS measurements were performed under the following operating conditions: RF power optimized for sensitivity, carrier gas flow rate of 0.38 L min^−1^, makeup gas flow rate of 0.65 L min^−1^, plasma gas flow of 15 L min^−1^, sampling depth of 10 mm, and a sample uptake rate of 0.35 mL min^−1^. The interface consisted of platinum sampling and skimmer cones. Ion lens voltages and quadrupole parameters were optimized using a 5 g L^−1^ tuning solution (Ce, Dy, Tm, and Lu in 2% (*v*/*v*) HCl). Quantification was performed using external calibration with certified multielement standard solutions prepared in the same acid matrix as the samples. An internal standard solution was continuously introduced to correct for instrumental drift and matrix effects during analysis. Instrument performance and calibration accuracy were verified following the analytical facility’s standard quality control procedures.

The concentration of active OH groups per unit area was calculated using the equation: C_OH_ = {(C_Zn_ × 10^−6^ × V × A)/(M × S)} × 2 where C_Zn_ = concentration of zinc ions, V = volume of nitric acid, S = surface area of specimens, A = Avogadro’s number, and M = molecular weight of zinc. All measurements were performed in triplicate to ensure reproducibility. To assess the chemical modifications occurring at each stage of the biofunctionalization process, the elemental and organic compounds present on the different surfaces were analyzed using Energy Dispersive X-ray Spectroscopy (EDS) (Zeiss Evo 10, Zeiss, Jena, Germany) and Fourier Transform Infrared Spectroscopy (FTIR, Spectrum Two, Perkin Elmer, Waltham, MA, USA). EDS spectra were collected at an accelerating voltage of 15 kV from at least three random regions per sample to ensure representativeness. FTIR analysis was performed in the range of 4000–400 cm^−1^ with a resolution of 4 cm^−1^ and 32 scans per spectrum.

### 4.4. In Vitro Cell Adhesion Assays

#### 4.4.1. Cell Culture and Biofunctionalized Specimen Preparation

Vero CCL-81 cells (ATCC), derived from the renal epithelium of the African green monkey (*Cercopithecus aethiops*) and widely used due to their stability and reproducible adherent behavior, were cultured in Dulbecco’s Modified Eagle Medium (DMEM, Gibco, Waltham, MA, USA) supplemented with 10% fetal bovine serum (FBS, Gibco) and 1% penicillin–streptomycin (100 U/mL and 100 μg/mL, respectively). Cultures were maintained at 37 °C in a humidified atmosphere containing 5% CO_2_.

For cell adhesion experiments, the biofunctionalized titanium specimens were disinfected with 96% ethanol for one hour and then air-dried under sterile conditions. Samples were then placed in 6-well plates, and 5 × 10^5^ cells in 4 mL of medium were seeded per well. After seeding, the plates were incubated at 37 °C for 24 h. All experiments were conducted in triplicate (*n* = 3) to ensure reproducibility.

#### 4.4.2. Cell Fixation, Staining, and Analysis

After the cell incubation period, the specimens were transferred to a new sterile plate and fixed with 4% formaldehyde for 10 min at room temperature. Subsequently, the specimens were rinsed three times with PBS, and cell nuclei were stained with 4′,6-diamidino-2-phenylindole (DAPI, 1:1000, Sigma-Aldrich, St. Louis, MO, USA) for 10 min in the dark [67]. The specimens were then rinsed with PBS and observed under a fluorescence microscope (Zeiss Axio Observer. Z1^®^, Jena, Germany). The number of cells was determined by counting nuclei in three randomly selected fields per specimen using ImageJ software (version 1.54).

#### 4.4.3. Statistical Analysis

All experiments were performed at least three times. Quantitative data were expressed as mean ± standard deviation (SD) when applicable. Statistical analysis for biological assays was performed using one-way or two-way analysis of variance (ANOVA), as appropriate, followed by Tukey’s multiple comparisons test, considering a significance level of *p* < 0.05. Elemental data obtained by EDS were used for qualitative and semi-quantitative surface comparison only and were not subjected to statistical inference.

## 5. Conclusions

The biofunctionalization of Ti6Al4V surfaces with *S. platensis* biomass and protein extract was successfully achieved, as evidenced by morphological, chemical, and biological analyses. Surface characterization by SEM revealed the presence of microalgal structures and protein deposits, with coverage patterns dependent on biomolecule concentration and immobilization time. EDS and FTIR analyses confirmed the chemical incorporation of organic elements (C, N, and the presence of phosphorus mainly associated with biomass-treated samples) consistent with the molecular composition of *S. platensis*, validating the immobilization process. An increase in oxygen, carbon and nitrogen related signals was observed in treated samples compared with the control, supporting the effective deposition of biological material onto the titanium surface. Differences in elemental composition were primarily associated with biomolecule type and concentration, reflecting variations in surface coverage and organic layer composition

Cell adhesion assays demonstrated a statistically significant enhancement in cell attachment to surfaces functionalized with *S. platensis* biomass, particularly at the highest tested concentration (5 g/L) and shorter immobilization times (3 h), which exhibited the highest cell density among all tested conditions. In contrast, longer immobilization times at the same concentration resulted in reduced cell adhesion, likely due to irregular surface coverage or decreased biomolecule availability, as supported by SEM observations. Protein-functionalized surfaces improved cell attachment compared with the unmodified control; their performance did not surpass that of optimally biomass-treated samples.

These findings underscore the distinct and complementary roles of biomass and protein extract in surface biofunctionalization. Biomass coatings provided a more complex and bioactive matrix, promoting both chemical signaling and topographical cues favorable to cellular interactions. Protein coatings contributed to nitrogen-rich surfaces that facilitated initial cell adhesion. The overall performance was strongly influenced by biomolecule type, concentration, and immobilization time, emphasizing the need to optimize these parameters to ensure reproducible and functional surface treatments.

It is important to emphasize that the biological performance discussed herein is based on early-stage cell adhesion and surface biocompatibility. Accordingly, the present study focused on early-stage cell adhesion as an indicator of surface bioactivity and did not aim to directly assess long-term osteointegration, which involves additional biological processes such as osteogenic differentiation, extracellular matrix deposition, and bone remodeling.

Therefore, future studies should incorporate additional in vitro assays, including cytotoxicity, cytomorphology, proliferation, differentiation and mineralization analyses, as well as in vivo models, to comprehensively evaluate the osteogenic potential of these coatings. Furthermore, the stability of microalgae-derived biomolecules during storage and their compatibility with clinically relevant sterilization methods should be systematically investigated to support the translational development of these biofunctionalized titanium surfaces.

## Figures and Tables

**Table 1:** Surface hydroxyl (OH) group concentration (C_OH_) of Ti6Al4V samples.

Sample	Zn Concentration (μg/L)	Zn Concentration (ppb)	Solution Volume (L)	Avogadro’s Number (L/mol)	Surface Area (nm^2^)	Molar Mass (g/mol)	C_OH_ (OH/nm^2^)	C_OH_ Normalized
Control	0.00255	3.61	0.06	6.02 × 10^23^	5.02 × 10^13^	62.75	827.88	1
Act 1:1	119.117	789.7	0.06	6.02 × 10^23^	5.02 × 10^13^	62.75	181,101.66	218.75
Act 3:1	0.22883	151.5	0.06	6.02 × 10^23^	5.02 × 10^13^	62.75	34,743.45	41.97

**Table 2:** EDS elemental composition of the base titanium surface and surfaces silanized for 1 and 3 h.

	Control		Sil1h		Sil3h	
	Weight %	Atomic %	Weight %	Atomic %	Weight %	Atomic %
Ti	88.97	84.70	78.55	63.52	79.17	58.06
Al	6.82	11.53	4.20	5.65	1.70	2.22
V	4.22	3.77	5.89	4.29	4.52	3.12
Si	0.00	0.00	0.55	0.72	0.56	0.70

**Table 4:** Elemental composition (EDS) of samples biofunctionalized with biomolecules.

	% Atomic	Ti	Al	V	C	Si	O	P	N
3 h	Biomass 1 g/L	58.83 ± 7.5	2.94 ± 1.2	4.27 ± 0.4	15.94 ± 2.8	0.21 ± 0.4	17.37 ± 5.1	0.43 ± 0.7	0
	Biomass 3 g/L	50.1 ± 22.2	4.19 ± 1.9	2.68 ± 2.6	11.05 ± 4.1	0.00	25.28 ± 22	4.96 ± 4.3	1.74 ± 3
	Biomass 5 g/L	52.54 ± 6.5	4 ± 0.1	3.79 ± 0.4	22.76 ± 3.1	0.64 ± 0.1	16.28 ± 3.3	0.00	0.00
	Protein 1 g/L	59.97 ± 16.7	4.77 ± 0.8	4.7 ± 1	18.23 ± 10	1.15 ± 1.1	3.3 ± 5.7	0.00	7.87 ± 4.6
	Protein 3 g/L	52.5 ± 2.8	4.65 ± 1	2.62 ± 2.3	24.34 ± 2.8	0.00	13.97 ± 3.8	0.00	1.88 ± 3.3
	Protein 5 g/L	57.45 ± 12.9	4.68 ± 1.7	2.27 ± 2	16.87 ± 5.5	0.00	5.02 ± 8.7	0.00	13.71 ± 7.4
14 h	Biomass 1 g/L	45.5 ± 6.9	3.7 ± 0.5	3.28 ± 0.5	29.78 ± 4.8	0.00	15.3 ± 2.9	2.46 ± 0.4	0.00
	Biomass 3 g/L	49.54 ± 2.1	4.06 ± 0.4	3.43 ± 0.2	25.95 ± 1.2	0.00	15.88 ± 1	1.14 ± 1	0.00
	Biomass 5 g/L	42.56 ± 19.6	2.92 ± 1.2	1.06 ± 1.8	26.64 ± 7.9	1.21 ± 1.1	23.75 ± 12.6	1.86 ± 1.6	0.00
	Protein 1 g/L	76.55 ± 1.8	6.67 ± 1.3	5.49 ± 0.7	8.73 ± 3.5	0.59 ± 1	0.00	0.00	1.97 ± 3.4
	Protein 3 g/L	61.19 ± 19.4	5.83 ± 1.3	3.42 ± 3	17.79 ± 15	0.71 ± 1.2	6.51 ± 11.3	0.00	4.55 ± 4.2
	Protein 5 g/L	64.2 ± 18.9	5.8 ± 2.6	4.3 ± 2	15.8 ± 4.8	0.00	5.6 ± 18.1	0.00	4.2 ± 3.9

## Data Availability

The datasets generated and/or analyzed during the current study are available from the corresponding author on reasonable request.

## Abbreviations

The following abbreviations are used in this manuscript:

Act 1:1	Surface activated with 1:1 piranha solution (H_2_SO_4_:H_2_O_2_)
Act 3:1	Surface activated with 3:1 piranha solution (H_2_SO_4_:H_2_O_2_)
ANOVA	Analysis of Variance
APTES	3-Aminopropyltriethoxysilane
ATR	Attenuated Total Reflectance
C_OH_	Concentration of active hydroxyl groups per unit area
C_Zn_	Concentration of zinc ions
DAPI	4′,6-diamidino-2-phenylindole
DES	Deep Eutectic Solvent
EDS	Energy Dispersive X-ray Spectroscopy
FBS	Fetal Bovine Serum
FTIR	Fourier Transform Infrared Spectroscopy
ICP-MS	Inductively Coupled Plasma Mass Spectrometry
M	Molar mass of zinc
PBS	Phosphate-Buffered Saline
S	Surface area of specimens
SD	Standard Deviation
SEM	Scanning Electron Microscopy
Sil1h	Surface silanized with APTES for 1 h
Sil3h	Surface silanized with APTES for 3 h
*S. platensis*	*Spirulina platensis*
TiB	Titanium functionalized with *S. platensis* biomass
TiP	Titanium functionalized with *S. platensis* protein extract
V	Volume of nitric acid
NA	Avogadro’s number
